# Should synovectomy be performed during total knee arthroplasty for knee osteoarthritis: A protocol for systematic review and meta-analysis

**DOI:** 10.1097/MD.0000000000027820

**Published:** 2021-11-19

**Authors:** Mingchao Li, Xiaoqing Shi, Songjiang Yin, Li Zhang, Peng Wu, Taiyang Liao, Lishi Jie, Peimin Wang

**Affiliations:** a Department of Orthopaedic Surgery, The Affiliated Hospital of Nanjing University of Chinese Medicine; 155 Hanzhong Road, Nanjing, Jiangsu, China; b Department of Orthopaedic Surgery, The Third People's Hospital of Kunshan, 615 Zizhu Road, Kunshan, Suzhou, Jiangsu, China.

**Keywords:** knee osteoarthritis, meta-analysis, pain, synovectomy, total knee arthroplasty

## Abstract

**Background:**

To evaluate the effect of synovectomy performed during primary total knee arthroplasty for knee osteoarthritis on patients’ postoperative pain and knee function.

**Methods:**

We will search the following electronic databases from inception to June 2021, including PubMed, EMBASE, Web of Science, the Cochrane Library, the China National Knowledge Infrastructure, the Chinese Scientific Journals Database, the Wanfang database, and the Chinese Biomedicine Database. Eligible references will be all randomized controlled trials of initial total knee arthroplasty for primary knee osteoarthritis with or without synovectomy. Two reviewers will independently extract the data. Reviewer Manager 5.3 software will be used for statistical analysis.

**Result:**

It will provide results on the short- and long-term efficacy and safety of synovectomy in total knee arthroplasty by various comprehensive assessments.

**Conclusion:**

This study will provide solid evidence on whether and when synovectomy treatment should be performed during total knee arthroplasty.

## 1 Introduction

Osteoarthritis is a common and disabling disease that currently affects 250 million people worldwide,^[1]^ and it is a significant personal, medical, and socioeconomic burden.^[2]^ Total knee arthroplasty (TKA) is an effective treatment for end-stage knee osteoarthritis (KOA).^[3]^ The overall satisfaction of TKA patients is high, but approximately 20% of patients are still dissatisfied with their results after surgery.^[4]^ Postoperative pain, infection, swelling, metallosis, and recurrent bleeding are the main causes of dissatisfaction. Synovectomy is frequently recommended or mentioned in the management of postoperative complications,^[5–8]^ as evidence indicates that synovectomy can resolve >90% of recurrent bleeding after TKA.^[9]^ Synovial inflammation is correlated with postoperative complications. On the one hand, surgical provocation of synovial tissue can cause inflammation, congestion, hyperplasia, and exudation; on the other hand, polyethylene abrasion and granulogenesis can lead to a synovial phagocytic cascade response, causing synovial proliferation, hypertrophy, and subsequent impingement, resulting in pain, swelling, or bleeding, thus prompting the clinician to wonder whether synovectomy be performed in KOA patients undergoing initial TKA.

Some researchers have suggested that synovectomy increases blood loss and operation time and has no beneficial effect on postoperative pain or function; thus, the synovium should be preserved as much as possible.^[10,11]^ Some studies have pointed out that synovectomy reduces sensory innervation of synovial tissue and reduces pain.^[12]^ Other studies have indicated that preoperative synovitis scores are negatively correlated with postoperative pain scores,^[13]^ to say that a milder preoperative synovitis can cause more postoperative pain. The reduction of postoperative pain is the key to achieving rapid recovery and improving patient satisfaction.^[14]^ However, there are no authoritative guidelines on whether to perform synovectomy in TKA, which mainly depends on the surgeon's intraoperative assessment of synovial health and surgical preference.^[15]^ Therefore, the question is raised concerning whether synovectomy is necessary in TKA. This question is very interesting and meaningful. Previous meta-analyses^[16–18]^ have reported the clinical outcomes of synovectomy in TKA, but there are some limitations, such as a small number of included studies, few outcome indicators, a short follow-up time, and a lack of randomized controlled trial (RCT) studies with a large sample size. An increasing number of clinical studies are emerging, and there is a need to re-evaluate the clinical outcomes of synovectomy performed in TKA and the scientific validity of the current RCT study design. Further consideration of the need for preoperative synovial inflammation assessment as a basis for intraoperative synovectomy is needed.

## 2 Methods

### 2.1 Protocol registration

This meta-analysis was registered on INPLASY, the ID was INPLASY2021100102 titled “Should synovectomy be performed during total knee arthroplasty for knee osteoarthritis: A protocol for systematic review and meta-analysis.” And this meta-analysis was performed according to the preferred reporting items for systematic reviews and meta-analyses 2020 checklist. This is a retrospective study and a protocol for meta-analysis, so the ethical approval was not required.

### 2.2 Inclusion criteria

Studies will be selected that met the following population, interventions, comparisons, outcomes, and study design (PICOS).

#### 2.2.1 Participants

The population met the diagnostic criteria for primary KOA with initial TKA.

#### 2.2.2 Interventions and comparisons

The synovectomy group underwent TKA with synovectomy, and the synovial preservation group underwent TKA without synovectomy.

#### 2.2.3 Outcomes

The outcome indicators included visual analog scale scores, blood loss, operation time, transfusion rate, knee society score clinical and functional scores, hospital for special surgery scores, joint mobility, and adverse events.

#### 2.2.4 Study designs

The RCTs evaluating the efficacy and safety of synovectomy during TKA for patients with KOA will be included, regardless of blinding.

### 2.3 Exclusion criteria

The exclusion criteria were as follows: research on nonprimary knee osteoarthritis; duplicate studies; review articles; literature with inappropriate statistical methods; basic research literature; animal studies; genetic studies; and grey literature.

### 2.4 Searching strategy

We will search synovectomy during TKA for end-stage KOA patients in PubMed (as shown in Table 1), EMBASE, Web of Science, the Cochrane Library, the China National Knowledge Infrastructure, the Chinese Scientific Journals Database, the Wanfang database, and the Chinese Biomedicine Database. We will also search for RCTs that are unpublished, including the International Clinical Trials Registry Platform, the NIH Clinical Trails, and the Chinese Clinical Register.

**Table 1:** Search strategy sample of PubMed.

No.	Search item
#1	Knee osteoarthritis[MeSH Terms]
#2	Knee osteoarthritis [Title/Abstract] OR KOA [Title/Abstract] OR osteoarthritis of knee [Title/Abstract]
#3	#1 OR #2
#4	total knee arthroplasty [MeSH Terms]
#5	“Arthroplasties, Replacement, Knee” [Title/Abstract] OR “Arthroplasty, Knee Replacement” [Title/Abstract] OR “Knee Replacement Arthroplasties” [Title/Abstract] OR “Replacement Arthroplasties, Knee” [Title/Abstract] OR “ Knee Arthroplasty, Total” [Title/Abstract] OR “ Arthroplasty, Total Knee” [Title/Abstract] OR “ Total Knee Arthroplasty” [Title/Abstract] OR “Replacement, Total Knee” [Title/Abstract] OR “Total Knee Replacement” [Title/Abstract] OR “Knee Replacement, Total” [Title/Abstract] OR “Arthroplasties, Knee Replacement” [Title/Abstract] OR “Replacement Arthroplasty, Knee” [Title/Abstract] OR “ TKA” [Title/Abstract] OR “TKR” [Title/Abstract]
#6	#4 OR #5
#7	synovectomy [MeSH Terms]
#8	“ Synovium Resection” [Title/Abstract] OR “ Resection, Synovium” [Title/Abstract] OR “ Synovium Resections ” [Title/Abstract] OR “ Synovectomies ” [Title/Abstract]
#9	#7 OR #8
#10	#3 AND #6 AND #9

### 2.5 Study selection and data extraction

#### 2.5.1 Selection of studies

**Figure 1:**
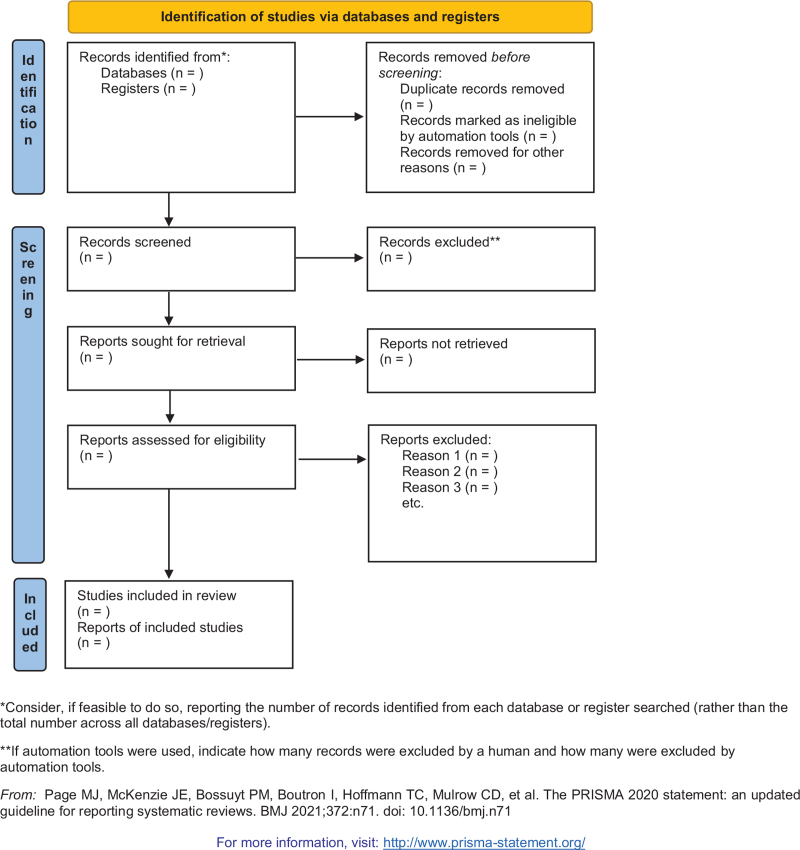
A PRISMA 2020 flow diagram of the literature screening and selection processes. PRISMA = preferred reporting items for systematic reviews and meta-analyses.

Note Express Version 3.2 will be used for literatures management. At first, duplicate documents will be deleted by software. Then 2 reviewers (ML and XS) will remove irrelevant articles independently by screening the titles and abstracts. If there is any uncertainty, we will obtain and read full texts. All the reasons for excluding studies will be recorded. A preferred reporting items for systematic reviews and meta-analysis flow chart has been drawn to illustrate the study selection procedure (Fig. 1)

#### 2.5.2 Data extraction and management

The eligible references will be retrieved and inserted into the Note Express. Two reviewers will independently retrieve and extract the data. The search language is limited to English and Chinese.

#### 2.5.3 Risk of bias in the included studies

According to the risk of bias assessment tool provided by Cochrane Reviewer's Handbook 5.2, 2 evaluators will assess the risk of bias independently, which included random sequence generation, allocation, concealment, blinding assessment, incomplete outcome data, reporting bias, and other biases. The risk of bias will be graded as low bias or unclear or high bias.

#### 2.5.4 Data synthesis and statistical analysis

Review Manager software Review Manager software (RevMan Version 5.3,

Copenhagen: The Nordic Cochrane Center, The Cochrane Collaboration, 2014) will be used to combine and calculate the outcomes. The results of the selected studies will be pooled for meta-analysis when ≥2 results are available. Continuous data will be entered as the means and standard deviations, and dichotomous outcomes are entered as the number of events. Continuous outcomes will be expressed as weighted mean differences and 95% confidence intervals. Dichotomous data will be stated as relative ratios and 95% confidence intervals.

#### 2.5.5 Assessment of heterogeneity

Heterogeneity will be detected by the X^2^ test, and if the heterogeneity among studies is small (*P* > .05, *I*^2^ < 50%), a fixed-effects model analysis will be used; if the heterogeneity is high (*P* ≤ .05, *I*^2^ ≥ 50%), a random-effects model analysis will be used; when *P* ≤ .05, *I*^2^ ≥ 50%, indicating the presence of statistical heterogeneity. Publication bias will be assessed using a funnel plot and Begger test.

#### 2.5.6 Subgroup analysis

The subgroup analysis will be carried out to explore possible reasons of the heterogeneity.

#### 2.5.7 Sensitivity analysis

The sensitivity analyses will be performed by removing the studies with high risk of bias in order to evaluate the stability of the results. To further confirm the stability of the above results, we excluded relevant studies sequentially for each outcome.

#### 2.5.8 Dealing with missing data

We will attempt to contact with the original authors via email or telephone to obtain the relevant information when there are missing data. If missing data cannot be obtained, we will still conduct an analysis using available data and assess the possible impact of missing data on the results by sensitivity analysis.

## 3 Discussion

With the ageing and obesity populations increasing, the number of KOA cases is rising and has become a significant public health burden. Pain is the main symptom of KOA and is also the main reason to choose TKA, as well as the main reason for postoperative dissatisfaction. Studies have found that synovial inflammation is associated with KOA pain.^[19]^ The synovium is the connective tissue membrane on the inner surface of the joint capsule and consists of 2 types of fibroblastic synovial cells: one located in the synovial lining layer, which is associated with inflammatory pain, swelling and disease progression; and the other located in the sub-synovial lining layer, which is associated with damage to bone and cartilage.^[20]^ Synovitis promotes the secretion of proinflammatory factors that aggravate the inflammatory response and activate nociceptive pain to initiate pain signals and even cause sensitization. Joint irrigation and synovectomy may reduce pain in patients with severe synovitis,^[12,21]^ decrease swelling, and increase mobility.^[22]^ Therefore, synovectomy may be beneficial in reducing postoperative pain and accelerating postoperative recovery during TKA.

There are many clinical studies on TKA, but few studies have compared the efficacy and safety of synovectomy during TKA. Therefore, the purpose of this study is to provide more convincing and detailed information for the synovectomy during TKA. We believe that the findings of this study will provide some recommendations to clinicians, for example, whether synovectomy should be performed, or which group of patients may benefit, or the advantages and disadvantages of synovectomy. This study will also be useful for clinicians to make preoperative surgery planning or design better evidence-based protocols in the future, and the patients with KOA will get benefit from this study.

## Acknowledgments

The authors would like to thank editors of American Journal Experts for useful corrections in this article.

## Author contributions

**Conceptualization:** Mingchao Li, Xiaoqing Shi, Peimin Wang.

**Data curation:** Mingchao Li, Li Zhang, Peng Wu, Taiyang Liao, Lishi Jie.

**Formal analysis:** Xiaoqing Shi, Songjiang Yin.

**Funding acquisition:** Mingchao Li, Peimin Wang.

**Investigation:** Xiaoqing Shi, Li Zhang, Peng Wu, Taiyang Liao, Lishi Jie.

**Methodology:** Mingchao Li, Xiaoqing Shi, Songjiang Yin, Li Zhang, Peng Wu, Peimin Wang.

**Project administration:** Peimin Wang.

**Resources:** Mingchao Li, Peimin Wang.

**Supervision:** Peimin Wang.

**Validation:** Mingchao Li, Li Zhang, Peng Wu, Taiyang Liao, Lishi Jie, Peimin Wang, Xiaoqing Shi.

**Writing - original draft:** Mingchao Li.

**Writing - review & editing:** Peimin Wang, Xiaoqing Shi.
